# A cross-validated deep learning framework for automated detection of DMBA-induced ovarian cancer from histopathological images with CA-125 biomarker support

**DOI:** 10.3389/frai.2026.1851807

**Published:** 2026-07-01

**Authors:** John Sushma Nannepaga, Kusuma Kandati, Munisankar Matam, Sai Lohitha Nayeneni, Megha Priya Adem

**Affiliations:** 1 Department of Biotechnology, Sri Padmavati Mahila Visvavidyalayam, Tirupati, India; 2 Department of Electronics and Communication Engineering, Sri Padmavati Mahila Visvavidyalayam, Tirupati, India; 3 Department of Computer Science and Engineering, Sri Padmavati Mahila Visvavidyalayam, Tirupati, India; 4 PM- USHA, R&D Cell, Sri Padmavati Mahila Visvavidyalayam, Tirupati, India

**Keywords:** artificial intelligence, CA-125, convolutional neural networks, DMBA-induced rat model, early diagnosis, histopathology, ovarian cancer

## Abstract

Ovarian cancer (OC) remains a major global health challenge due to its asymptomatic progression and the lack of reliable diagnostic tools. Recent advances in artificial intelligence (AI) offer promising opportunities to enhance diagnostic accuracy through automated analysis of biomedical images and integration of multimodal data. Female albino rats were divided into control and cancer–induced groups. OC were chemically induced by 7,12-Dimethylbenz[a]anthracene (DMBA) exposure. Blood samples were collected at baseline and predefined post-induction time points to quantify serum Cancer Antigen 125 (CA-125) levels, which were significantly elevated in the cancer group (438.7 ± 6.4 U/mL) compared with controls (22.5 ± 3.1 U/mL; *p* < 0.01). Ovarian tissues were harvested, processed, and imaged for high-resolution histopathological analysis. A custom convolutional neural network (CNN) was developed to classify ovarian tissue images into normal and cancerous categories. The dataset consisted of 904 labeled images and was evaluated using stratified data splitting and 5-fold cross-validation. The proposed CNN achieved a test accuracy of 96.69%, precision of 95.8%, recall of 97.2%, and F1-score of 96.5%. Cross-validation further demonstrated robust and stable performance with a mean accuracy of 96.42 ± 0.52%. Comparative benchmarking showed superior performance over conventional machine learning methods and transfer learning models, including Support Vector Machine, Random Forest, MobileNetV2, ResNet50, and EfficientNetB0. These findings indicate that the integration of serum biomarkers, histopathology, and deep learning provides a robust preclinical framework for ovarian cancer detection. This AI-assisted diagnostic strategy demonstrates strong translational potential for improving early diagnosis, risk stratification, and clinical decision support in ovarian cancer.

## 1 Introduction

Ovarian cancer poses a significant global health challenge, marked by high mortality rates and a lack of reliable diagnostic methods in its early stages (Ren et al., 2025). The asymptomatic nature of early ovarian cancer often leads to diagnosis at advanced stages, when treatment is less effective and survival rates are significantly lower (Hormaty et al., 2025). Therapeutic intervention is most effective in the early stages, where the five-year survival rate can reach 95%. However, approximately 70–75% of ovarian cancer cases are diagnosed at advanced stages (III or IV), where the five-year survival rate drops to below 30% (Atallah et al., 2021). Biomarkers have emerged as crucial tools for the early detection, risk stratification, and prognostication of ovarian cancer (Salar et al., 2025). Although CA125 is the most extensively studied biomarker for ovarian cancer, it lacks specificity and can be elevated in benign conditions such as endometriosis, pelvic inflammatory disease and menstruation. Other biomarkers include HE4, CA19-9, CEA, AFP, microRNAs, circulating tumor DNA, and autoantibodies against TP53 and *β*-hCG are also used either alone or in combination depending on the ovarian cancer subtype (Bindu et al., 2024; Zhang et al., 2022). Current screening methods, such as serum CA125, transvaginal sonography and MRI are often insufficient due to limited sensitivity and specificity, highlighting the urgent need for improved approaches (Bast Jr et al., 2020). All surgically resected tissue specimens are routinely submitted for histopathological evaluation, which serves as the gold standard for definitive diagnosis and classification of ovarian cancer subtypes (Orsulic et al., 2022).

Conventional diagnostic methods rely on histopathological analysis (Wu et al., 2023) and imaging techniques, which require expert interpretation and are prone to subjectivity. To overcome these limitations, computer-aided diagnosis (CAD) systems have been increasingly explored. Initial research efforts focused on traditional machine learning techniques using handcrafted features extracted from ovarian tissue images. Texture descriptors such as gray-level co-occurrence matrices (GLCM), wavelet features, and statistical measures were combined with classifiers including support vector machines (SVM) and k-nearest neighbors (KNN) (Wen et al., 2014; Verma and Kumar, 2025). Although these methods improved diagnostic accuracy compared to manual assessment, their performance heavily depended on feature engineering and domain knowledge.

The emergence of deep learning significantly transformed medical image analysis. Convolutional neural networks (CNNs) demonstrated superior performance by automatically learning hierarchical features from raw images (Mallya et al., 2025). Recent studies emphasize the integration of molecular biomarkers with computational diagnostic frameworks to enhance ovarian cancer detection accuracy (Ronsini et al., 2024). Krizhevsky et al. (2012) highlighted the effectiveness of deep CNNs in image classification, which inspired their adoption in biomedical applications (Mienye et al., 2025). Subsequently, CNNs were successfully applied to cancer detection tasks such as breast cancer (Wang et al., 2025) lung cancer (Kashyap et al., 2025), and skin cancer (Esteva et al., 2017).

Several studies have applied CNN-based models specifically to ovarian cancer detection using histopathological and microscopic images. Arevalo et al. (2016) demonstrated that deep learning approaches outperform traditional methods in identifying malignant tissue patterns. Transfer learning using pre-trained CNN architectures has also been employed to address limited dataset availability, resulting in improved classification performance (Tajbakhsh et al., 2016; Elkenawy et al., 2024). The novelty of the present study lies in the development of a biologically validated multimodal ovarian cancer diagnostic framework that integrates CA-125 biomarker analysis, histopathological imaging, and a custom CNN model using a DMBA-induced preclinical rat model, with superior performance over conventional machine learning and state-of-the-art transfer learning methods. This study provides a reproducible and efficient preclinical diagnostic pipeline with strong translational potential for clinical application in ovarian cancer.

## 2 Materials and methods

### 2.1 Experimental design and grouping

**Figure 1:**
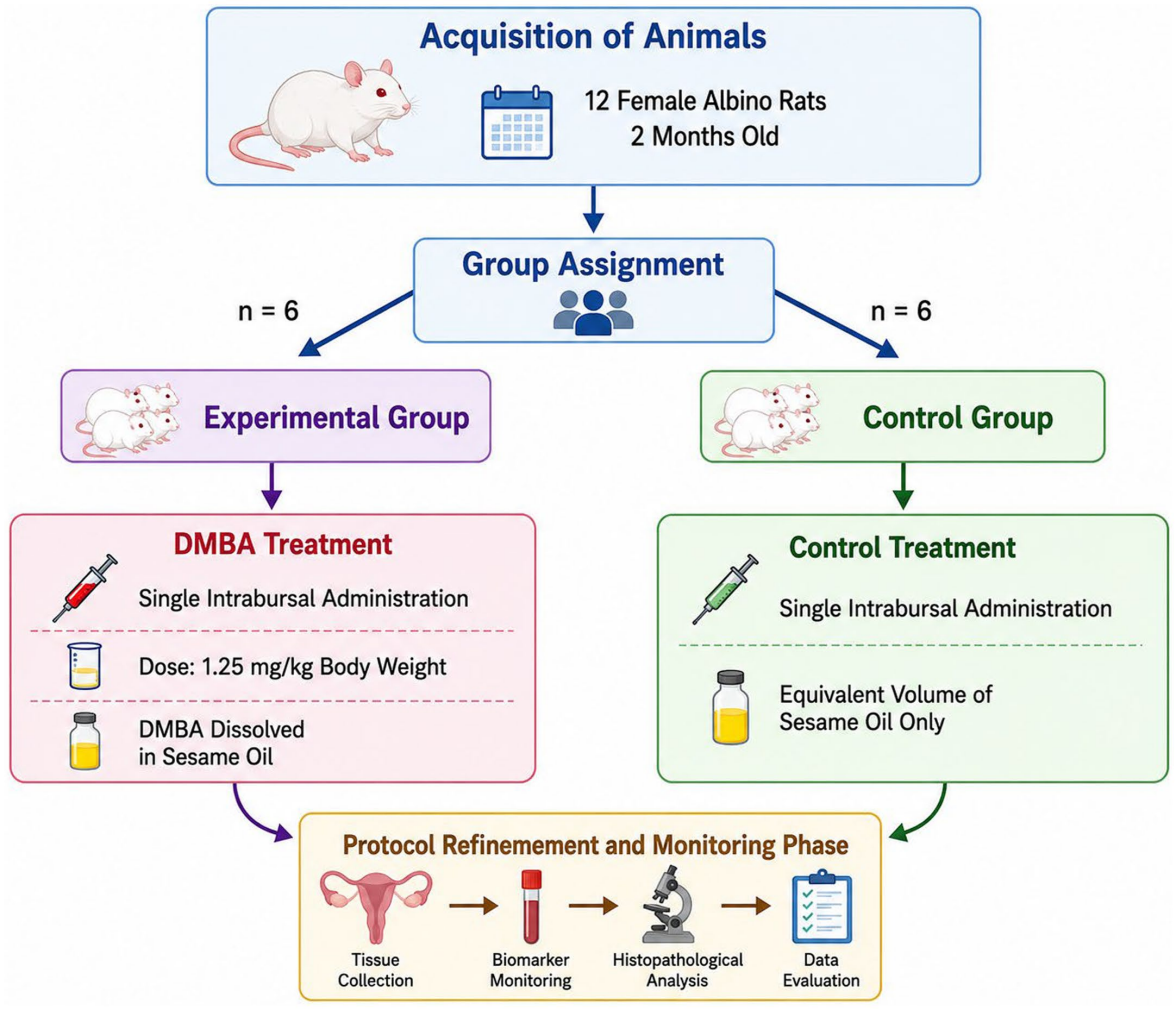
Experimental design for DMBA-induced ovarian cancer model in albino rats.

All experimental procedures involving albino rats were conducted in accordance with the guidelines of the Institutional Animal Ethics Committee (IAEC) of Sri Padmavati Mahila Visvavidyalayam, Tirupati, India (Approval No. CPCSEA/1677/SPMVV/IAEC/III-07, dated 10-03-2023). A total of 12 healthy female albino rats, 2 months old and weighing 150–200 g, were procured from Sri Venkateswara Enterprises, Bangalore. Animals were housed in sanitized polycarbonate cages under standard laboratory conditions: temperature 24 ± 2 °C, relative humidity 45–64%, and a 12:12 h light/dark cycle. Rats received a standard commercial pellet diet and water ad libitum via nipple-fitted plastic bottles. Animals were randomly allocated into two experimental groups. With six rats in each group (*n* = 6) as shown in Figure 1.

Group I: Healthy control.

Group II: Ovarian cancer–induced group.

### 2.2 Induction of ovarian cancer

Ovarian cancer was induced using the chemical carcinogen 7,12-dimethylbenz[a]anthracene (DMBA). Rats in the ovarian cancer group received a single intrabursal administration of DMBA at a dose of 1.25 mg/kg body weight, dissolved in sterile sesame oil (Goncalves et al., 2025). Following carcinogen administration, animals were maintained for 90 days to allow tumor initiation and progression. Rats in the control group received no carcinogen treatment. At the end of the experimental period, blood samples were collected from the animals. The rats used in the study were within the acceptable body weight range (<200 g) for cervical dislocation. To avoid possible chemical contamination that could interfere with histopathological analysis, no anesthesia was used. Animals were humanely euthanized by cervical dislocation performed by trained personnel under the supervision of a veterinarian, in accordance with the IAEC-approved protocol (Approval No. CPCSEA/1677/SPMVV/IAEC/III-07) and institutional ethical guidelines. Following euthanasia, ovarian tissues were immediately excised and fixed in 10% neutral buffered formalin to preserve morphological integrity and prevent degradation. The preserved tissues were subsequently processed for histopathological evaluations. Successful induction of ovarian tumors was confirmed by gross morphological observations and histopathological examination of ovarian tissues.

### 2.3 Blood sample collection

Blood samples were collected at baseline and at predefined time points during the post-induction period. Samples were allowed to clot at room temperature and centrifuged at 2000 rpm for 10 min at 4 °C to separate serum (Hoyer et al., 2009). Serum samples were stored at −20 °C until analysis. Serum levels of the ovarian cancer biomarker carbohydrate antigen 125 (CA-125) were quantified using commercially available Enzyme-Linked Immunosorbent Assay (ELISA) kits according to the manufacturer’s instructions. Changes in CA-125 levels were used to monitor tumor development and progression.

### 2.4 Tissue collection

At the end of the experimental period, animals were euthanized, and ovarian tissues were excised, fixed in 10% neutral buffered formalin, dehydrated through graded ethanol series, cleared, and embedded in paraffin wax. Sections of 5 μm thickness were prepared using a rotary microtome and stained with hematoxylin and eosin (H&E) (Slaoui et al., 2017). The stained sections were examined under a light microscope to assess morphological changes such as follicular disruption, stromal invasion, cyst formation, cellular atypia, necrosis, and inflammatory infiltration, which are characteristic features of ovarian cancer.

### 2.5 Image acquisition and dataset preparation

**Figure 2:**
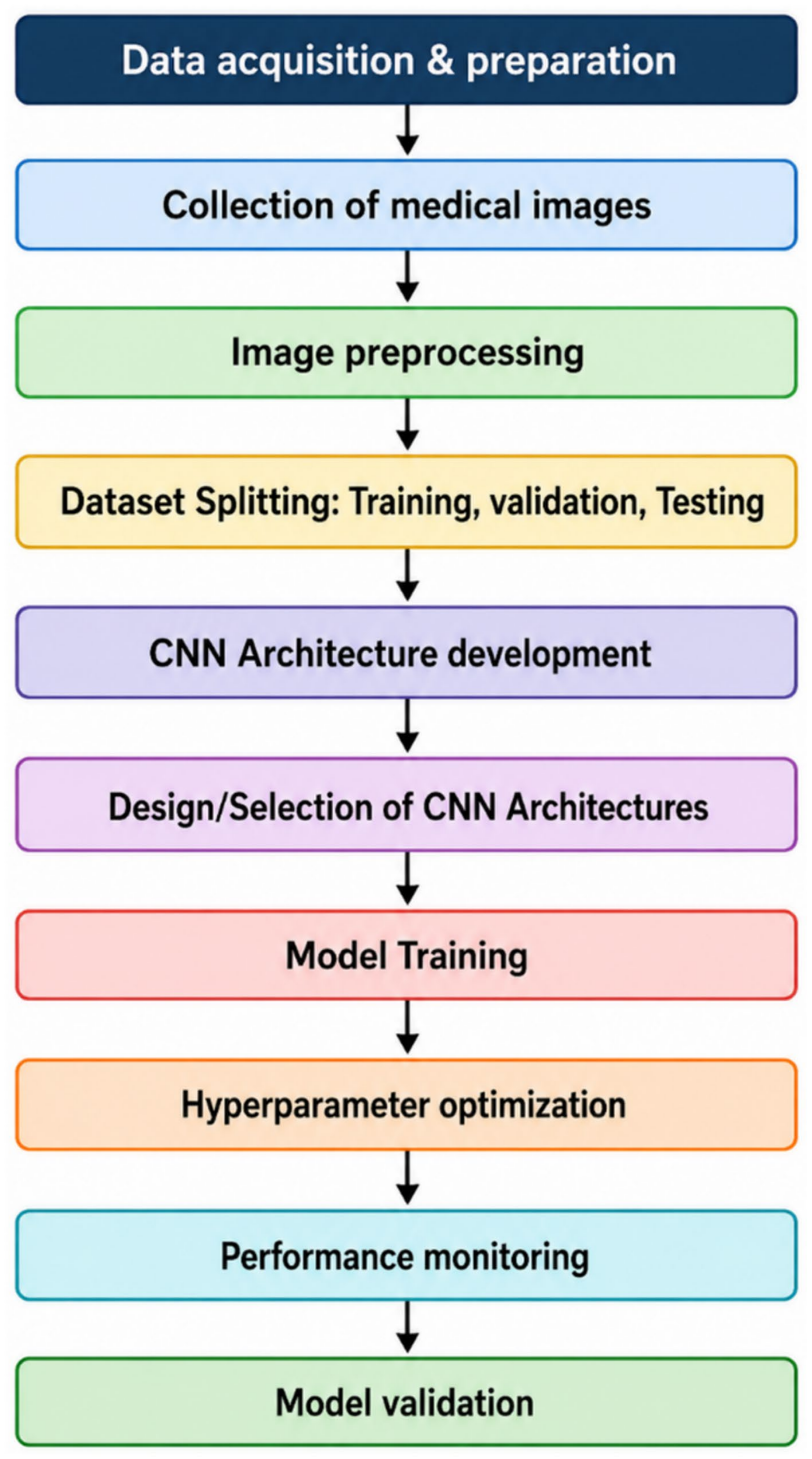
Workflow of the deep learning–based ovarian cancer classification pipeline.

The dataset employed in this study consists of microscopic ovarian tissue images used for the automated detection of ovarian cancer. All histopathological images used in this study were obtained exclusively from ovarian tissue sections of DMBA-induced albino rats generated during the experimental procedure. No human patient datasets were included. Histopathological images were captured using a digital microscope under standardized illumination and magnification settings (Breen et al., 2025). The resulting dataset formed the basis for deep learning-based image analysis as depicted in Figure 2.

### 2.6 Dataset composition

The final dataset consisted of 904 labeled histopathological images belonging to two classes:

- Normal ovarian tissue
- Cancerous ovarian tissue

The dataset was partitioned into training, validation, and testing subsets using stratified random sampling to preserve class balance and reduce sampling bias. Approximately equal numbers of image samples were obtained from each animal to minimize animal-specific bias.

Dataset distribution:

- Training set: 723 images
- Testing set: 181 images

To improve model robustness and reduce overfitting, multiple microscopic fields were extracted from each tissue sample to capture histological heterogeneity.

#### 2.6.1 Overall system architecture

The proposed system follows a supervised deep learning approach to classify ovarian tissue images into normal and cancerous categories (Gülmez, 2026). Initially, microscopic ovarian images are acquired and pre-processed to enhance quality and uniformity. The processed images are then fed into a CNN model, which automatically extracts hierarchical features and performs classification. The final output indicates the presence or absence of ovarian cancer.

#### 2.6.2 Image preprocessing

**Figure 3:**
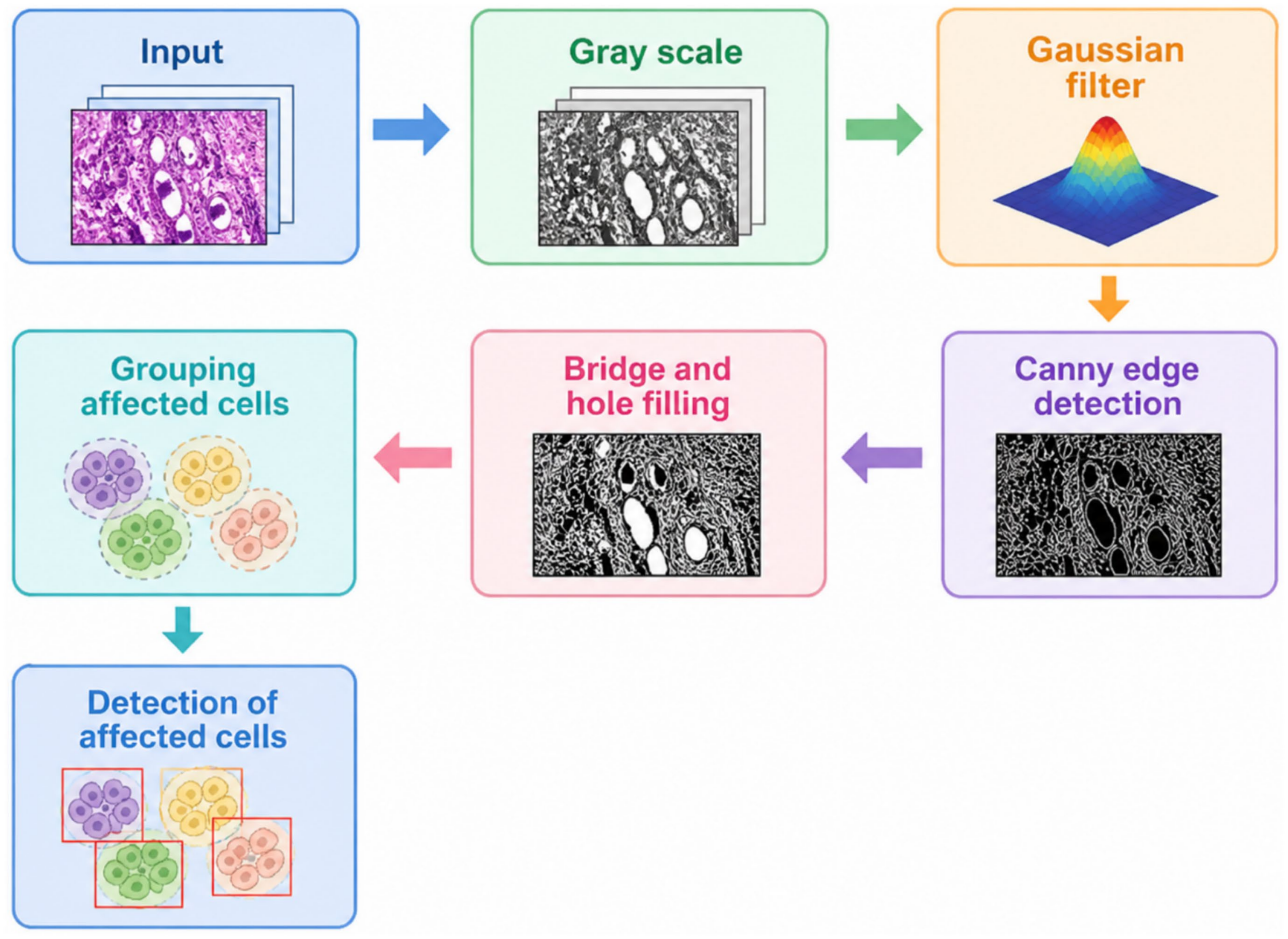
Steps involved in image preprocessing.

To ensure uniformity and improve model generalization, all histopathological images underwent a standardized preprocessing pipeline:

Resizing: Images were resized to a fixed spatial resolution of 224 × 224 pixels to ensure compatibility with the CNN input layer.
Normalization: Pixel intensity values were normalized to the range [0,1] to stabilize gradient updates during training.
Noise and Artifact Reduction: Low-quality images containing staining artifacts or illumination inconsistencies were excluded to enhance feature clarity.
Color Standardization: RGB color space consistency was maintained across samples to preserve histological texture information (see Figure 3).

##### 2.6.2.1 Pre-processing

Firstly, the biological RGB cell image (bitmap image) is converted into grey-scale image. A pre-processing technique is applied to grey-scale image to improve the quality of image and also to eliminate the useless information using Gaussian Filter.

##### 2.6.2.2 Gaussian filter

In a Gaussian filter is rolled over the cytology image to smoothen the region of interest. Filter the image with isotropous Gaussian smoothing kernels of increasing standard deviations. Gaussian filters are isotropic with the same standard deviation along both dimensions. An image can be filtered by an isotropic Gaussian filter by specifying a scalar value for sigma.

##### 2.6.2.3 Canny edge detection algorithm

Edge function is used to find edges in an intensity image. The Canny edge detection is a multi-stage algorithm which is used to detect a wide range of edges in cell images. The Canny edge technique is used to extract the abrupt changes of affected cells and non- affected cells. This technique helps us to make the difference between affected and non affected cells.

##### 2.6.2.4 Morphological operations

Morphological functions perform morphological operation on gray-scale image to detect the affected cells.

- Bridge: bridges previously unconnected pixels in the gray-scale image.
- Close: performs morphological closing operation (dilation operation followed by erosion)
- Open: performs morphological opening operation (erosion followed by dilation).
- Erode: performs erosion using the structuring element.
- Imfill: morphological function fills image regions and holes.
- Imdilate: morphological function dilates the gray-scale image and returns the dilated image.

##### 2.6.2.5 Grouping affected cells

Adaptive thresholding is an image segmentation algorithm that appears quite resistant to varying lighting conditions. The most basic thresholding method is to choose a fixed threshold value and compare each pixel to that value.

The affected cells will be retained by using adaptive threshold technique. The maximum intensity values of smoothen image is considered as background.

##### 2.6.2.6 Extraction of holes

Where, affected cells forms large sized holes and non-affected cells forms small sized holes. Some of the cells could not generate the holes, at that time bridge morphological function is used to generate the holes.

##### 2.6.2.7 Extraction of affected cells

**Figure 4:**
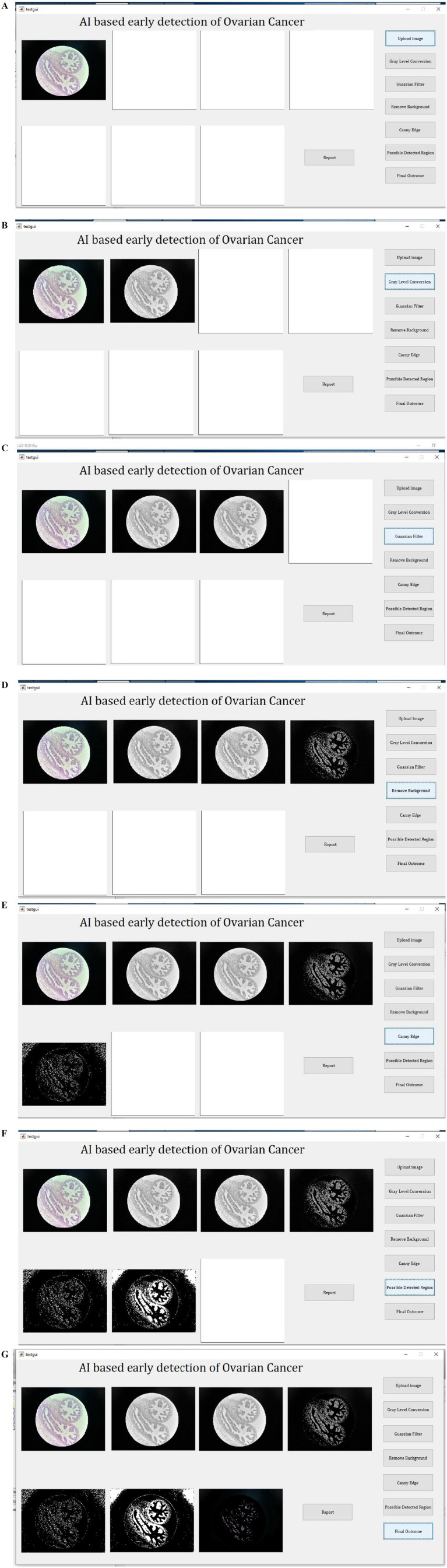
**(A)** Upload of an image. **(B)** Gray level conversion. **(C)** Images after applying Gaussian filter. **(D)** Results after background removal. **(E)** Results after applying Canny Edge. **(F)** Results after possible detected region. **(G)** Final outcome.

The larger components are considered as affected cells. To retain these larger components, the morphological functions are used effectively. The Figures 4A–G shows the various steps involved in image processing for the dataset.

#### 2.6.3 CNN architecture design

The proposed CNN model comprises four convolutional blocks, followed by fully connected layers and a softmax classifier. Each convolutional block consists of:

A convolutional layer with 3 × 3 kernels.

ReLU activation function to introduce non-linearity.

Max-pooling (2 × 2) for spatial downsampling.

The detailed architecture is as follows:

1. Input Layer: Preprocessed ovarian tissue image
2. Conv Block 1: 32 filters → ReLU → Max Pooling
3. Conv Block 2: 64 filters → ReLU → Max Pooling
4. Conv Block 3: 128 filters → ReLU → Max Pooling
5. Conv Block 4: 256 filters → ReLU → Max Pooling
6. Flatten Layer: Converts feature maps into a 1D vector
7. Fully Connected Layer: 256 neurons with ReLU activation
8. Dropout Layer (0.5): To reduce overfitting
9. Output Layer: Softmax activation for binary classification

This architecture enables progressive learning of low-level features (edges, textures) and high-level morphological patterns relevant to ovarian cancer pathology.

#### 2.6.4 Model training and optimization

Model training was performed using the Adam optimizer, chosen for its adaptive learning rate and fast convergence properties. The following hyperparameters were used:

Loss function: Categorical Cross-Entropy.

Optimizer: Adam.

Learning rate: 0.001.

Batch size: 32.

Number of epochs: 50.

To mitigate class imbalance, data augmentation techniques such as rotation, horizontal flipping, and zooming were applied to the training set. Model performance was monitored using the validation set, and early stopping was employed to prevent overfitting.

##### 2.6.4.1 Cross-validation strategy

To ensure robustness and reduce sampling bias, a 5-fold cross-validation strategy was employed. The complete dataset was randomly divided into five equal subsets. In each iteration, four subsets were used for training and one subset for validation/testing. This process was repeated five times so that each subset served once as the validation set. Final performance metrics were reported as the mean ± standard deviation across all folds.

#### 2.6.5 Performance evaluation metrics

The trained CNN model was evaluated on an unseen test set using standard classification metrics:

- Accuracy
- Precision
- Recall
- F1-score
- Confusion matrix

The metric definitions were:

$$Accuracy=\frac{TP+TN}{TP+TN+FP+FN}$$

$$Precision=\frac{TP}{TP+FP}$$

$$Recall=\frac{TP}{TP+FN}$$

$$F1=2\times \frac{Precision\times Recall}{Precision+Recall}$$

Where TP, TN, FP, and FN denote true positives, true negatives, false positives, and false negatives, respectively.

#### 2.6.6 Implementation environment

The deep learning framework was implemented using MATLAB Deep Learning Toolbox on a GPU-accelerated computing environment. GPU acceleration significantly reduced training time and enabled efficient handling of high-dimensional image data.

### 2.7 Statistical analysis

All experimental data were expressed as mean ± standard deviation (SD). Statistical comparisons between control and ovarian cancer groups were performed using Student’s t-test. A *p*-value less than 0.05 was considered statistically significant.

## 3 Results

### 3.1 Induction and confirmation of ovarian cancer

**Figure 5:**
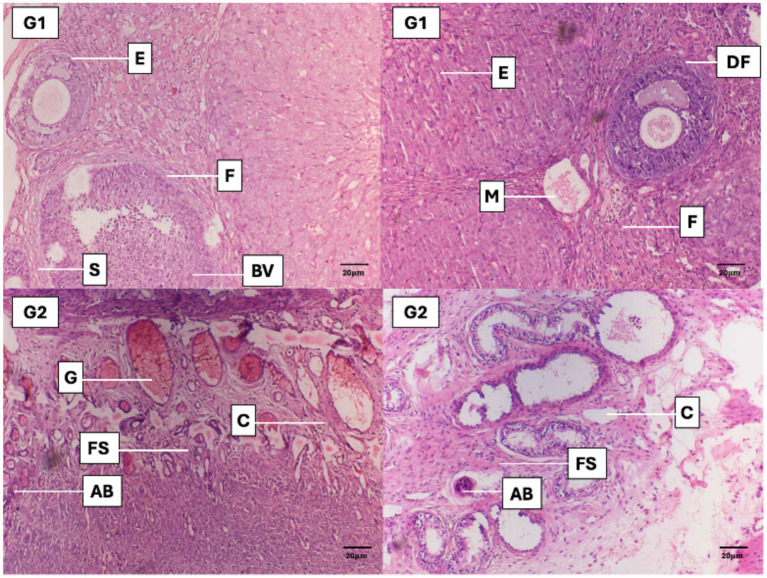
Representative H&E-stained ovarian sections showing normal morphology in control rats (G1) and severe structural alterations in DMBA-induced ovarian cancer rats (G2).

Induction of ovarian cancer was achieved in the experimental group following DMBA administration, as evidenced by gross morphological changes and histopathological confirmation. Rats exposed to DMBA developed visibly enlarged, irregular, and nodular ovaries compared to the smooth and compact ovaries observed in control animals. These macroscopic alterations are consistent with previous reports validating DMBA as an effective chemical carcinogen for inducing ovarian tumors in rat models. Histopathological examination further confirmed tumor development in DMBA-treated rats. Ovarian tissue sections revealed disrupted architecture, stromal invasion, cellular pleomorphism, hyperchromatic nuclei, cystic formations, and increased mitotic activity. In contrast, control ovarian tissues displayed intact follicles at various developmental stages, well-organized stroma, and absence of inflammatory or neoplastic changes as shown in Figure 5. These findings confirm reliable tumor induction and provide a robust pathological basis for subsequent diagnostic analyses. Tumor tissues exhibited loss of follicular integrity, extensive stromal remodeling, neovascularization, inflammatory cell infiltration, and regions of necrosis. The presence of cystic and glandular structures suggested epithelial-origin malignancy. In contrast, ovaries from control rats retained normal histoarchitecture with clearly defined follicles, corpus luteum, and stromal organization. The sharp contrast between groups highlights the reliability of histopathological diagnosis as a gold standard reference and provided high-quality labeled data for training and evaluating AI-based image analysis models.

### 3.2 Serum CA-125 biomarker analysis

Serum levels of CA-125 were significantly elevated in the ovarian cancer group compared to controls. A progressive increase in CA-125 was observed at post-induction time points, indicating tumor progression and active disease state. Control rats maintained consistently low CA-125 levels throughout the study period as represented in Table 1. The elevation of CA-125 in tumor-bearing rats supports its utility as a diagnostic biomarker in this preclinical model. Although CA-125 is known to have limitations in early-stage human ovarian cancer due to low specificity, its significant rise in chemically induced tumors highlights its relevance when combined with histopathological and imaging data.

**Table 1:** Serum CA-125 levels in control and DMBA-induced ovarian cancer groups.

Group	Control (mean ± SD)	DMBA (mean ± SD)	*p*-value
CA-125 (U/mL)	22.5 ± 3.1	438.7 ± 6.4	<0.01

Data are expressed as mean ± standard deviation (SD). Statistical significance was determined by appropriate comparative analysis between groups. A *p*-value < 0.05 was considered statistically significant.

### 3.3 Performance evaluation metrics of the model

The proposed CNN model demonstrated strong classification performance in distinguishing normal and cancerous ovarian histopathological images (Table 2).

**Table 2:** Performance evaluation metrics of the model.

Metric	Value (%)
Accuracy	96.69
Precision	95.8
Recall	97.2
F1-Score	96.5

The model achieved an overall classification accuracy of 96.69%, indicating reliable predictive capability. A precision of 95.8% reflects a low false-positive rate, while the recall of 97.2% demonstrates high sensitivity in detecting cancerous tissues. The F1-score of 96.5% confirms balanced performance between precision and recall. Collectively, these results indicate that the proposed CNN architecture provides robust and consistent classification of ovarian histopathological images.

### 3.4 Confusion matrix analysis

**Figure 6:**
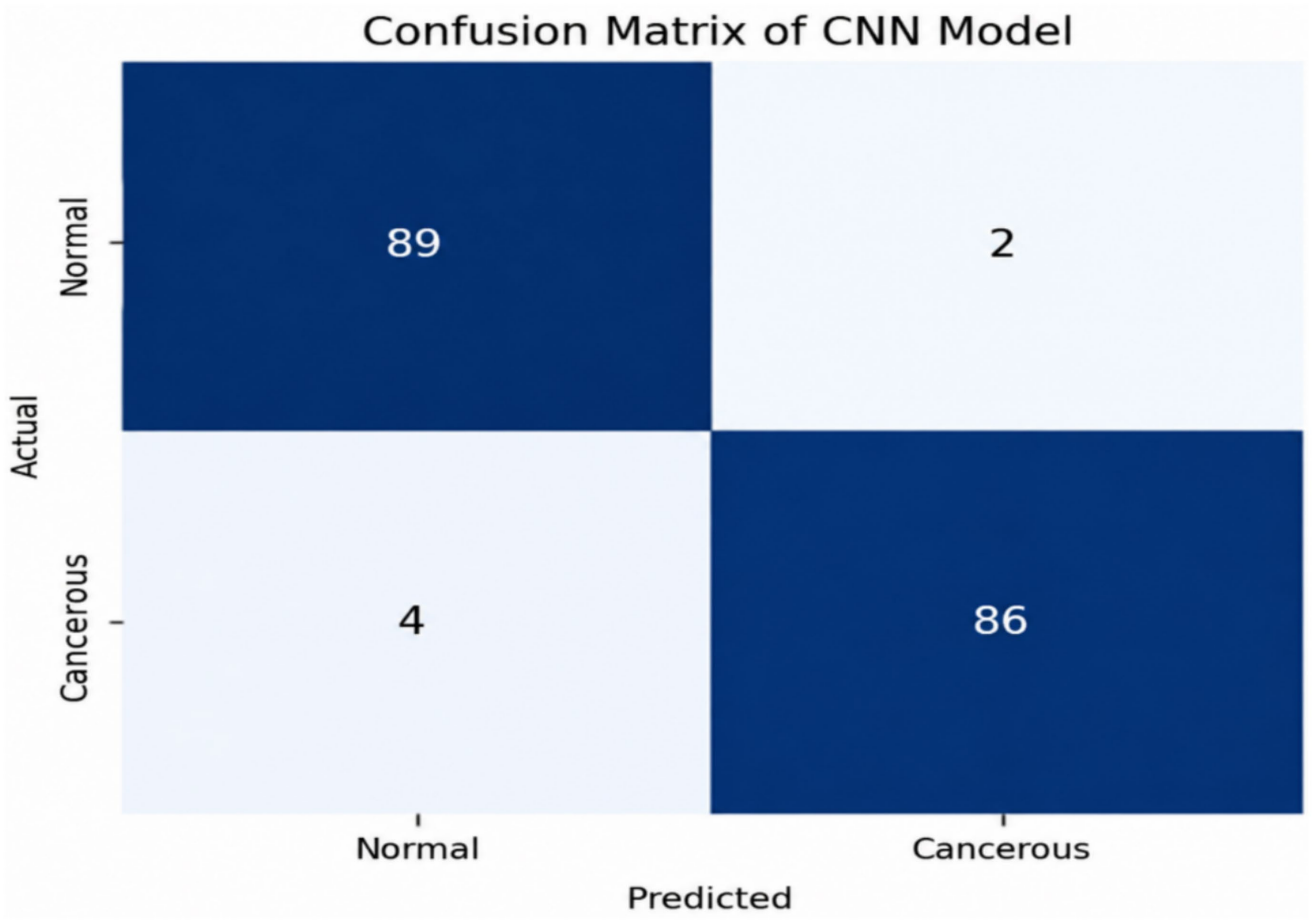
Confusion matrix of proposed CNN model.

The confusion matrix presented in Figure 6 demonstrates the strong classification performance of the proposed CNN model in distinguishing normal and cancerous ovarian tissue images. Out of the total 181 samples, the model correctly classified 175 samples, resulting in an overall accuracy of approximately 96.69%. Specifically, among the 91 normal samples, 89 were correctly identified as normal, while only 2 samples were incorrectly classified as cancerous. Similarly, among the 90 cancerous samples, 86 were correctly classified as cancerous, whereas 4 samples were misclassified as normal.

The low number of false positives (2 cases) indicates that the model has high specificity and effectively avoids misclassifying healthy tissues as malignant. Likewise, the limited false negatives (4 cases) demonstrate strong sensitivity, showing that the model successfully detected the majority of cancerous tissues. These results confirm that the proposed CNN model achieved balanced and reliable predictive performance with minimal diagnostic error. The confusion matrix further highlights the robustness of the model for automated ovarian cancer detection and supports its potential application in computer-aided histopathological diagnosis.

### 3.5 Training convergence analysis

Training and validation curves demonstrated stable learning behaviour across 50 epochs. The training and validation accuracy curves showed a progressive increase with only a minimal gap between them, indicating effective learning and satisfactory generalization performance. Similarly, both training and validation loss curves decreased consistently throughout training, confirming convergence without substantial overfitting. These observations suggest that the proposed regularization strategy, including dropout and augmentation, effectively improved model stability.

**Figure 7:**
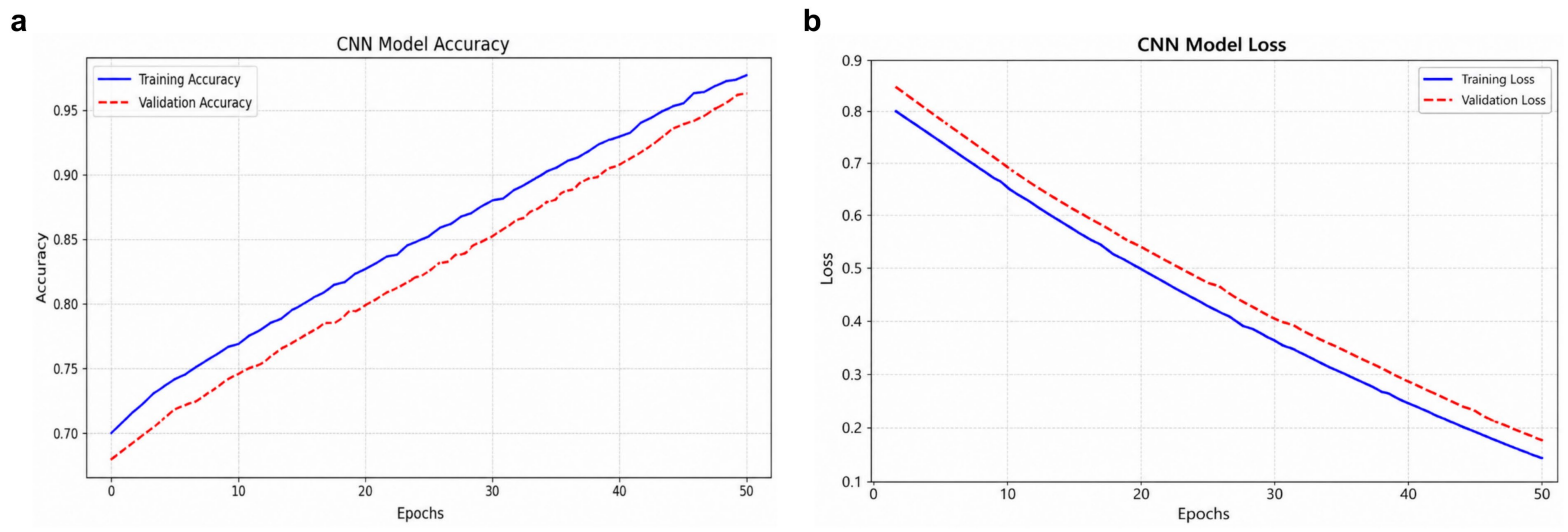
**(A)** Training and validation accuracy for the CNN model, showing convergence after 50 epochs. **(B)** Training and validation loss curves for the CNN model, showing convergence after 50 epochs.

The convergence trend observed in Figures 7A,B suggests effective model generalization with minimal overfitting. The figure depicts the progression of training and validation accuracy over successive epochs. Both curves show a consistent upward trend with a minimal gap between them, indicating effective learning and good generalization performance of the proposed CNN model. The reduction in training and validation loss over successive epochs. Both curves demonstrate a consistent downward trend with a small gap between them, indicating stable model convergence and effective generalization without significant overfitting.

### 3.6 Five-fold cross-validation results

To assess robustness and reduce split-dependent bias, five-fold cross-validation was performed (see Table 3).

**Table 3:** Five-fold cross-validation results.

Fold	Accuracy (%)	Precision (%)	Recall (%)	F1-score (%)
Fold 1	95.8	95.1	96.2	95.6
Fold 2	96.4	95.7	96.8	96.2
Fold 3	97.1	96.5	97.5	97.0
Fold 4	96.0	95.4	96.6	96.0
Fold 5	96.8	96.1	97.0	96.5
Mean ± SD	96.42 ± 0.52	95.76 ± 0.54	96.82 ± 0.47	96.26 ± 0.50

The low standard deviation across folds indicates stable generalization and suggests that model performance was not dependent on a single random data split.

### 3.7 Comparative performance analysis

To evaluate the added value of the proposed CNN framework, its performance was quantitatively compared with representative traditional machine learning classifiers and alternative deep learning models using the same dataset and evaluation protocol. For traditional approaches, handcrafted texture features were extracted from histopathological images and classified using Support Vector Machine (SVM) and k-Nearest Neighbors (KNN) classifiers. In addition, a baseline shallow CNN architecture was implemented for comparison with the proposed deep CNN model. All models were trained and tested using identical data splits to ensure a fair and unbiased comparison. Performance was evaluated using accuracy, precision, recall, and F1-score (see Table 4).

**Table 4:** Comparative performance of different classification methods.

Method	Accuracy (%)	Precision (%)	Recall (%)	F1-score (%)
KNN + texture features	88.2	87.5	86.9	87.2
SVM + texture features	90.4	89.8	90.1	89.9
Baseline CNN	93.1	92.4	92.8	92.6
Random forest + texture features	91.8	91.2	90.9	91.0
ResNet50	95.2	94.8	95.5	95.1
EfficientNetB0	95.8	95.3	96.0	95.6
MobileNetV2	94.5	94.0	94.2	94.0
XG Boost + texture features	94.2	93.7	94.0	93.8
Proposed CNN	96.69	95.8	97.2	96.5

The proposed CNN model outperformed both conventional machine learning classifiers and state-of-the-art transfer learning architectures. While EfficientNet-B0 and ResNet-50 demonstrated strong classification performance, the proposed architecture achieved the highest recall and overall accuracy, indicating superior sensitivity in detecting ovarian cancer tissues. This suggests that the custom architecture effectively captured domain-specific morphological patterns while maintaining computational efficiency.

### 3.8 Computational complexity analysis of proposed CNN model

The proposed CNN model consists of four convolutional blocks, followed by fully connected layers and a softmax classifier for binary ovarian tissue image classification. The computational complexity of the model is primarily dominated by convolution operations, while pooling and activation layers contribute relatively less overhead.

1 Complexity of Convolutional Layers

For a convolutional layer, the computational complexity is given by:

$$O\left(H\times W\times K^{2}\times C_{in}\times C_{out}\right)$$ (1)

Where:

-
H,W = height and width of feature map
-
K = kernel size (3 × 3)
-
$C_{in}$ = number of input channels
-
$C_{out}$ = number of output filters

Since the proposed model uses four convolutional blocks with increasing filters (32, 64, 128, 256), the total complexity becomes:

$$O\left(\sum_{l=1}^{4}H_{l}W_{l}K^{2}C_{in}^{\left(l\right)}C_{out}^{\left(l\right)}\right)$$ (2)

The complexity of one convolutional layer is given in Equation 1, while the total network complexity is shown in Equation 2.

2 Complexity of Pooling Layers

The computational complexities associated with pooling operations, fully connected layers, and memory requirements are described in Equation 3–5.

Each max-pooling layer performs down sampling with 2 × 2 windows:

$$O\left(H\times W\right)$$ (3)

Pooling reduces spatial dimensions, thereby lowering subsequent convolution costs.

3 Complexity of Fully Connected Layer

After flattening, the dense layer with 256 neurons has complexity:

$$\;O\left(N\times 256\right)$$ (4)

Where N is the flattened input size.

4 Output Layer Complexity

For binary classification using softmax:

$$O\left(256\times 2\right)$$

which is negligible compared to convolutional layers.

Thus, although the proposed model requires higher training time than traditional machine learning methods such as KNN or SVM, it provides significantly improved feature extraction capability and superior classification performance.

#### 3.8.1 Space complexity

The memory requirement depends on:

- Trainable weights of convolutional and dense layers
- Intermediate feature maps during forward/backward propagation

Thus, space complexity is proportional to:

$$O\left(P+F\right)$$ (5)

Where:

-
P = total trainable parameters
-
F = stored feature maps

The use of max-pooling after each convolution block progressively reduces feature map size, helping control computational cost despite increasing filter numbers. Therefore, the proposed CNN achieves a good trade-off between computational efficiency and classification accuracy (96.69%), making it suitable for automated ovarian cancer diagnosis. Compared with conventional methods, the proposed CNN incurs moderate computational overhead during training but offers fast inference and substantially improved diagnostic accuracy, justifying its practical deployment in clinical image analysis.

## 4 Discussion

The present findings clearly demonstrate the successful induction of ovarian cancer in albino rats following DMBA administration, as evidenced by the marked elevation of the tumor biomarker CA-125 (Goncalves et al., 2025). In the control group, CA-125 levels remained within the physiological range (22.5 ± 3.1 U/mL), reflecting normal ovarian function and the absence of pathological changes. In contrast, the DMBA-treated group exhibited a dramatic increase in CA-125 levels (438.7 ± 6.4 U/mL), with the difference being statistically significant (*p* < 0.01) (Sandhiutami et al., 2019). This pronounced elevation strongly indicates tumor development and validates the effectiveness of DMBA as a chemical carcinogen for inducing ovarian cancer in experimental rat models. The narrow standard deviation in the DMBA group further suggests consistent tumor induction across animals, highlighting the reliability and reproducibility of the experimental model (Charkhchi et al., 2020). These results support the use of serum CA-125 as a sensitive indicator for confirming ovarian tumor development in preclinical studies. Importantly, the clear distinction between control and cancer groups establishes a robust baseline for subsequent investigations involving histopathological evaluation and integration with advanced diagnostic approaches such as deep learning-based image analysis. CA125 levels observed in the DMBA-induced rat model reflect experimental tumor progression and should not be directly equated with clinical staging categories in human ovarian cancer. The integration of histopathology with deep learning enabled automated detection of morphological patterns associated with malignancy. The proposed CNN model achieved strong predictive performance, with an accuracy of 96.69%, precision of 95.8%, recall of 97.2%, and F1-score of 96.5%. These findings indicate that the model effectively distinguished normal and cancerous ovarian tissues with minimal diagnostic error. Particularly, the high recall is clinically relevant because reducing false-negative predictions is essential in cancer screening systems, where missed detections may delay treatment initiation (Li et al., 2025).

The confusion matrix analysis further demonstrated balanced classification behaviour, with only two false-positive and four false-negative predictions. The low false-positive rate suggests that the model is unlikely to over diagnose healthy tissue, whereas the limited false negatives indicate strong sensitivity toward malignant tissue recognition. Such balance is desirable for computer-aided pathology systems intended for decision support.

Five-fold cross-validation further strengthened the reliability of the findings. The low standard deviation across folds suggests that model performance was stable and not dependent on a single random train-test split. This indicates acceptable generalization within the available dataset and supports the reproducibility of the proposed architecture.

Comparative analysis showed that the proposed CNN outperformed conventional machine learning models such as SVM, KNN, Random Forest, and XGBoost, as well as modern transfer learning networks including MobileNetV2, ResNet50, and EfficientNetB0. This result suggests that a carefully designed domain-specific CNN may outperform larger generic pre-trained networks when trained on specialized histopathological datasets. The custom architecture likely benefited from learning task-specific morphological representations while maintaining lower computational complexity.

From a computational perspective, the proposed model achieved a favourable trade-off between performance and efficiency. Although deep learning models generally require greater training resources than conventional machine learning methods, inference after training is rapid and suitable for automated screening workflows. This makes the framework practical for future deployment in digital pathology environments.

Despite these promising findings, several limitations should be acknowledged. Firstly the present study utilizes a well-established DMBA-induced ovarian cancer rat model, which provides a controlled and reproducible platform for evaluating disease progression. However, further validation using human clinical samples would strengthen the translational applicability of the proposed framework. The study focuses on CA-125 as a clinically relevant biomarker, which is widely used in ovarian cancer diagnosis. Incorporating additional complementary biomarkers in future studies may further enhance diagnostic precision and robustness of the multimodal approach. Third, only binary classification (normal vs. cancerous) was considered, whereas real clinical scenarios require subclassification of tumor grade, stage, and histological subtype.

Future studies should therefore focus on validating the framework using large-scale multi-institutional human histopathological datasets. Incorporation of explainable AI techniques such as Grad-CAM, multimodal fusion with biomarkers and radiology, and subtype-specific classification may further enhance translational relevance.

Overall, this study demonstrates that integrating experimental oncology models with deep learning-based histopathological analysis can produce accurate and efficient ovarian cancer detection systems. The findings support the growing role of AI in precision pathology and provide a strong preclinical foundation for future human translational research.

## 5 Conclusion

These findings highlight the diagnostic relevance of CA-125 as a sensitive biomarker for confirming ovarian cancer development in preclinical studies. The clear differentiation between healthy and cancer-induced groups provides a strong foundation for integrating histopathological evaluation and advanced computational approaches, such as deep learning-based image analysis, to enhance early detection strategies. The proposed CNN-based framework effectively automates ovarian cancer detection by integrating preprocessing, augmentation, deep feature extraction, and classification. This methodology minimizes human intervention while achieving reliable diagnostic performance, making it suitable for computer-aided clinical decision support systems. Overall, this study contributes valuable insights into ovarian cancer diagnosis and establishes a validated preclinical proof-of-concept platform for future translational research. Although promising, the proposed AI-based diagnostic pipeline requires large-scale validation using human clinical datasets before translation into routine clinical practice aimed at improving early diagnostic accuracy and clinical outcomes.

## Data Availability

The original contributions presented in the study are included in the article/supplementary material, further inquiries can be directed to the corresponding author.
